# 2-(6-Hydroxyhexylthio)-5,8-dimethoxy-1,4-naphthoquinone Induces Apoptosis through ROS-Mediated MAPK, STAT3, and NF-*κ*B Signalling Pathways in Lung Cancer A549 Cells

**DOI:** 10.1155/2020/7375862

**Published:** 2020-08-12

**Authors:** Gui-Nan Shen, Cheng Wang, Ying-Hua Luo, Jia-Ru Wang, Rui Wang, Wan-Ting Xu, Yi Zhang, Yu Zhang, Dong-Jie Zhang, Cheng-Hao Jin

**Affiliations:** ^1^Department of Biochemistry and Molecular Biology, College of Life Science & Technology, Heilongjiang Bayi Agricultural University, Daqing, Heilongjiang 163319, China; ^2^Pharmacy Department, Daqing Oilfield General Hospital, Daqing 163001, China; ^3^Department of Grass Science, College of Animal Science & Veterinary Medicine, Heilongjiang Bayi Agricultural University, Daqing, Heilongjiang 163319, China; ^4^Department of Food Science and Engineering, College of Food Science & Technology, Heilongjiang Bayi Agricultural University, Daqing, Heilongjiang 163319, China; ^5^National Coarse Cereals Engineering Research Center, Daqing, Heilongjiang 163319, China

## Abstract

Two novel compounds, 2-(2-hydroxyethylthio)-5,8-dimethoxy-1,4-naphthoquinone (HEDMNQ) and 2-(6-hydroxyhexylthio)-5,8-dimethoxy-1,4-naphthoquinone (HHDMNQ), were synthesized to investigate the kill effects and mechanism of 1,4-naphthoquinone derivatives in lung cancer cells. The results of the CCK-8 assay showed that HEDMNQ and HHDMNQ had significant cytotoxic effects on A549, NCI-H23, and NCI-H460 NSCLC cells. Flow cytometry and western blot results indicated that HHDMNQ induced A549 cell cycle arrest at the G2/M phase by decreasing the expression levels of cyclin-dependent kinase 1/2 and cyclin B1. Fluorescence microscopy and flow cytometry results indicated that HHDMNQ could induce A549 cell apoptosis, and western blot analysis showed that HHDMNQ induced apoptosis through regulating the mitochondria pathway, as well as the MAPK, STAT3, and NF-*κ*B signalling pathways. Flow cytometry results showed that intracellular reactive oxygen species (ROS) levels were increased after HHDMNQ treatment, and western blot showed that ROS could modulate the intrinsic pathway and MAPK, STAT3, and NF-*κ*B signalling pathways. These effects were blocked by the ROS inhibitor N-acetyl-L-cysteine in A549 cells. Our findings suggest that compared with HEDMNQ, HHDMNQ had the stronger ability to inhibit the cell viability of lung cancer cells and induce apoptosis by regulating the ROS-mediated intrinsic pathway and MAPK/STAT3/NF-*κ*B signalling pathways. Thus, HHDMNQ might be a potential antitumour compound for treating lung cancer.

## 1. Introduction

Lung cancer mainly occurs in the bronchial epithelium, and it causes the most cancer-related deaths worldwide [1]. Non-small-cell lung cancer (NSCLC) is a highly metastatic and aggressive lung cancer subtype, accounting for about 80% of lung cancer cases [2]. Standard therapies include surgery, chemotherapy, and molecular-targeted therapy, but the mortality rate for lung cancer patients is still high [3–5]. Therefore, it is highly desirable to find more effective novel therapeutic targets and develop drugs with high efficacy and low toxicity for NSCLC patients.

Mitogen-activated protein kinases (MAPKs), such as extracellular signal-regulated kinase (ERK), c-Jun N-terminal kinase (JNK), and p38, are involved in cancer cell proliferation, migration, and apoptosis [6–8]. The nuclear factor-*κ*B (NF-*κ*B) signalling pathway includes NF-*κ*B and nuclear factor of kappa light polypeptide gene enhancer in B-cells inhibitor, alpha (I*κ*B-*α*), and has a crucial role in the regulation of cell survival and apoptosis [9]. Signal transducer and activator of transcription 3 (STAT3) is involved in the activation of immunity and apoptosis, and its activation depends on cytokines and growth factors [10]. In addition, some studies have demonstrated that the phosphorylation of MAPKs is associated with activation of the NF-*κ*B signalling pathway [11]. Furthermore, NF-*κ*B and STAT3 are constitutively activated in lung cancer and are promising targets for the development of novel cancer drugs [12–14]. Thus, the MAPK, STAT3, and NF-*κ*B signalling pathways may be key targets for cancer therapy.

Reactive oxygen species (ROS) are produced by mitochondrial oxidative phosphorylation and play crucial roles as intracellular messengers in cell proliferation, differentiation, and survival [15]. Oxidative stress can induce excessive production of ROS, leading to cellular damage and apoptosis [16]. Furthermore, ROS play essential roles in maintaining various biological functions by modulating many signal transduction pathways [17]. Therefore, controlling the levels of ROS is important for the treatment of cancer.

The 1,4-naphthoquinone derivatives have been exploited in drug development because of their high anticancer activity and low cytotoxicity [18]. Thus, the anticancer effects of the 1,4-naphthoquinone compound have been the focus of recent studies [18–20]. The pharmacophore of 1,4-naphthoquinone imparts anticancer activity in a number of drugs, such as menadione, shikonin, and doxorubicin. The 1,4-naphthoquinone derivatives have been synthesized from natural terpenoids and p-benzoquinones, followed by side chain transformation. Several studies have shown that 1,4-naphthoquinone derivatives with substituent groups at the C-2 site can promote antiproliferative activity [21–23].

In this study, we synthesized two novel naphthoquinone derivatives, 2-(2-hydroxyethylthio)-5,8-dimethoxy-1,4-naphthoquinone (HEDMNQ) and 2-(6-hydroxyhexylthio)-5,8-dimethoxy-1,4-naphthoquinone (HHDMNQ). Then, we investigated the effects of HEDMNQ and HHDMNQ on cell viability, cell cycle, cell apoptosis, apoptosis-related signalling pathways, and intracellular ROS levels in A549 lung cancer cells.

## 2. Materials and Methods

### 2.1. Synthesis of HEDMNQ and HHDMNQ

Aluminum chloride (1.06 M) and sodium chloride (0.484 M) were heated to 150°C, followed by the addition of 1,4-dimethoxybenzene (0.12 M) and maleic anhydride (0.24 M). The mixture was incubated at 170–180°C for 5–10 min. After cooling, 1800 mL of double distilled water (DDW) and 120 mL of 98% hydrogen chloride were added to the mixture, with constant stirring for 12–18 h. Then, naphthazarin was obtained by recrystallizing the filter residue. Naphthazarin (100 mM), butyraldehyde (200 mL), sodium hydrosulfite (60 mM), DDW (200 mL), and tetrabutylammonium bromide (2 g) were stirred for 2 h at room temperature, and a mixture of sodium dithionite (10.6 g), 50% sodium hydroxide solution (40 mL), and dimethyl sulfate (25 mL) was added every 3 h. After constant stirring for 12–18 h, 1,4,5,8-tetramethoxynaphthalene was generated and dissolved in acetonitrile (450 mL) and chloroform (150 mL). Ceric ammonium nitrate (54 g) was added to the solution and stirred for 0.5–1 h, and 5,8-dimethoxy-1,4-naphthoquinone was obtained by recrystallizing. Next, 2-hydroxy-1-ethanethiol (1.5 mM) and 6-mercapto-1-hexanol (1.5 mM) were added to a solution of 5,8-dimethoxy-1,4-naphthoquinone (1 mM) in MeOH (30 mL). The mixture was stirred for 4 h at room temperature and stirred for 5 min with sodium dichromate (0.6 mM) and sulfuric acid (0.6 mM). Two novel compounds, HEDMNQ and HHDMNQ, were separated by chromatography. Nuclear magnetic resonance (NMR) spectra were recorded on a JNM-AL 400 (400 MHz) spectrometer. Chemical shifts (d) are given in ppm downfield from tetramethylsilane as the internal standard. Mass spectra were collected with the AB SCIEX API 2000 LC/MS/MS (Applied Biosystems, Inc., Foster City, CA, USA) and LCMS-IT-TOF (Shimadzu Scientific Inc., Beijing, China) systems.

### 2.2. Cell Culture

Human lung cancer cell lines A549, NCI-H23, and NCI-H460 were obtained from the American Type Culture Collection (Manassas, VA, USA); and normal lung IMR-90, liver L-02, and stomach GES-1 cell lines were purchased from Saiqi Biological Engineering Co., Ltd. (Shanghai, China). The cells were cultured in Dulbecco's Modified Eagle's Medium (DMEM) supplemented with 10% fetal bovine serum (FBS) and grown at 37°C in a humidified atmosphere with 5% CO_2_.

### 2.3. Cell Counting Kit-8 Assay Determination of Cell Viabilities

Briefly, the human lung cancer A549, NCI-H23, and NCI-H460 cells and normal lung IMR-90, liver L-02, and stomach GES-1 cell lines were counted and seeded into 96-well culture plates followed by adherence for 24 h. Then, the cells were treated with different concentrations of shikonin, fluorouracil (5-FU), HEDMNQ, and HHDMNQ (0, 1, 3, 10, 30, and 100 *μ*M) for 24 h. Followed by starvation for 2 h, 10 *μ*L of CCK-8 solution (Beyotime Biotechnology, Beijing, China) was added to each well. After incubation for 1 h at 37°C, the absorbance of the solution was measured at 450 nm with an automatic microplate reader (BioTek Instruments, Inc., Winooski, VT).

In addition, the A549, NCI-H23, NCI-H460, IMR-90, L-02, and GES-1 cells were seeded in 96-well plates. According to the IC_50_ value of shikonin, 5-FU, HEDMNQ, and HHDMNQ in human lung cancer and normal cell lines, the cells were treated with HEDMNQ and HHDMNQ for 3, 6, 12, 24, and 36 h. Next, 10 *μ*L of CCK-8 solution was added to the cells and measured by using an automatic microplate reader.

### 2.4. Flow Cytometry Determination of Cell Cycle

A549 cells were incubated with 6 *μ*M HHDMNQ for the indicated times (3, 6, 12, and 24 h), and the cells were harvested and fixed in 70% ethanol at 4°C for 24 h. Cell suspensions were resuspended in 195 *μ*L binding buffer and incubated with RNase and propidium iodide (PI) (Beyotime Biotechnology) for 30 min in the dark at 37°C. Then, the cells were analyzed by flow cytometry (Beckman Coulter, Brea, CA, USA) with a 488 nm argon laser.

### 2.5. Fluorescence Microscopy Determination of Apoptosis

Briefly, 4 × 10^4^ cells/well of A549 cells were cultured onto 6-well plates followed by adherence overnight and subsequent treatment with 6 *μ*M HHDMNQ for the indicated times (3, 6, 12, and 24 h). Then, the cells were harvested and suspended in phosphate-buffered saline (PBS), after which the cell suspensions were stained with Hoechst 33342 and PI (Beyotime Biotechnology) for 15 min in the dark and analyzed by fluorescence microscopy (EVOS FL, Life technology, USA).

### 2.6. Flow Cytometry Determination of Apoptosis

A549 cells were incubated with 6 *μ*M HHDMNQ for the indicated times (3, 6, 12, and 24 h), and the cells were harvested and resuspended in PBS. Then, the cell suspensions were stained with Annexin V-FITC and PI (Beyotime Biotechnology) for 15 min in the dark and analyzed by flow cytometry (Beckman Coulter).

### 2.7. Flow Cytometry Determination of ROS

Many studies have shown that DCFH-DA staining is one of the most widely used techniques for directly measuring the redox state of cells. A549 cells were incubated with 6 *μ*M HHDMNQ for the indicated times (3, 6, 12, and 24 h), and incubated with 10 *μ*M 2′,7′-dichlorofluorescein diacetate (DCFH-DA) (Merck, Shanghai, China) for 30 min at 37°C. Then, the cells were centrifuged at 5,000 ×*g* for 5 min, washed, and resuspended in PBS. 2′,7′-Dichlorofluorescein fluorescence was analyzed by flow cytometry.

### 2.8. Western Blot Analysis of Proteins

A549 cells were harvested and lysed in lysis buffer. The supernatant was collected after centrifugation at 12,000 ×*g* for 30 min at 4°C and dissolved at 30 *μ*g proteins per 20 *μ*L with DDW and 5× buffer. The proteins were separated on 10% SDS-PAGE gels and electrotransferred onto nitrocellulose membranes. Then, the membranes were blocked in 5% skim milk and incubated with primary antibodies overnight at 4°C. After incubation with secondary antibodies, the protein bands were detected using enhanced chemiluminescence detection reagents. The intensity of the bands was determined using the ImageJ software.

### 2.9. Statistical Analysis

The results were analyzed by comparison between different groups. The IC_50_ was calculated by the GraphPad Prism 5.0 software. The continuous data were analyzed by one-way analysis of variance followed by Tukey's post-hoc tests using the SPSS 18.0 statistical software. Statistically significant values were defined as *p* < 0.05. All of the experiments were replicated three times and presented as mean ± standard deviation (SD).

## 3. Results

### 3.1. Synthesis of the 1,4-Naphthoquinone Derivatives HEDMNQ and HHDMNQ

**Figure 1:**
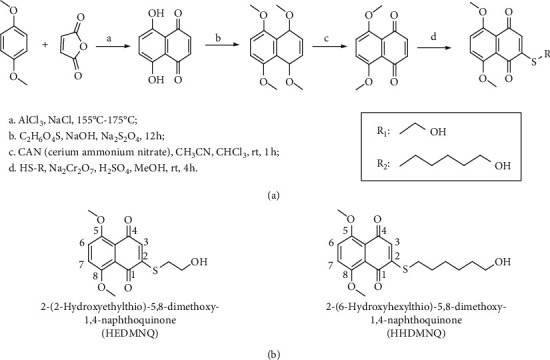
Synthesis of 1,4-naphthoquinone derivatives HEDMNQ and HHDMNQ. (a) Reagents and conditions of HEDMNQ and HHDMNQ. (b) Chemical structures of HEDMNQ and HHDMNQ.

To improve anticancer activity and reduce the side effects of 1,4-naphthoquinone, we synthesized HEDMNQ and HHDMNQ (Figure 1). By performing NMR at a wavelength of 400 MHz, we analyzed the H, C and mass spectra in deuterated chloroform solvent and identified the following structures.

HEDMNQ : ^1^H-NMR (CDCl_3_, 400 MHz): *δ* 7.34 (d, *J* = 9.6 Hz, 1H), 7.28 (d, *J* = 9.6 Hz, 1H), 6.61 (s, 1H), 3.96 (s, 6H), 3.93 (t, *J* = 6.4 Hz, 2H), 3.05 (t, *J* = 6.4 Hz, 2H); ^13^C NMR (CDCl_3_, 150 MHz): *δ* 181.8 (C-1), 181.6 (C-4), 154.3 (C-5), 153.6 (C-8), 153.5 (C-2), 127.8 (C-3), 123.3 (C-7), 121.0 (C-6), 120.7 (C-10), 119.7 (C-9), 56.8 (OCH_3_), 56.8 (OCH_3_), 59.7 (C-1′), 31.9 (C-2′); *m*/*z* 316.9 (*M* + Na)^+^.

HHDMNQ : ^1^H-NMR (CDCl_3_, 400 MHz): *δ* 7.33 (d, *J* = 9.6 Hz, 1H), 7.27 (d, *J* = 9.6 Hz, 1H), 6.45 (s, 1H), 3.96 (s, 6H), 3.66 (t, *J* = 6.4 Hz, 2H), 2.76 (t, *J* = 7.6 Hz, 2H), 1.78–1.72 (m, 2H), 1.61–1.25 (m, 6H); ^13^C NMR (CDCl_3_, 150 MHz): *δ* 181.76 (C-1), 181.74 (C-4), 154.4 (C-5), 154.3 (C-8), 153.7 (C-2), 127.5 (C-3), 121.0 (C-7), 120.91 (C-6), 120.91 (C-10), 119.6 (C-9), 56.8 (OCH_3_), 56.5 (OCH_3_), 56.9 (C-1′), 32.3 (C-2′), 31.9 (C-3′), 31.4 (C-4′), 30.4 (C-5′), 30.2 (C-6′); *m*/*z* 372.9 (*M* + Na)^+^.

### 3.2. HEDMNQ and HHDMNQ Inhibit Lung Cancer Cell Proliferation

**Figure 2:**
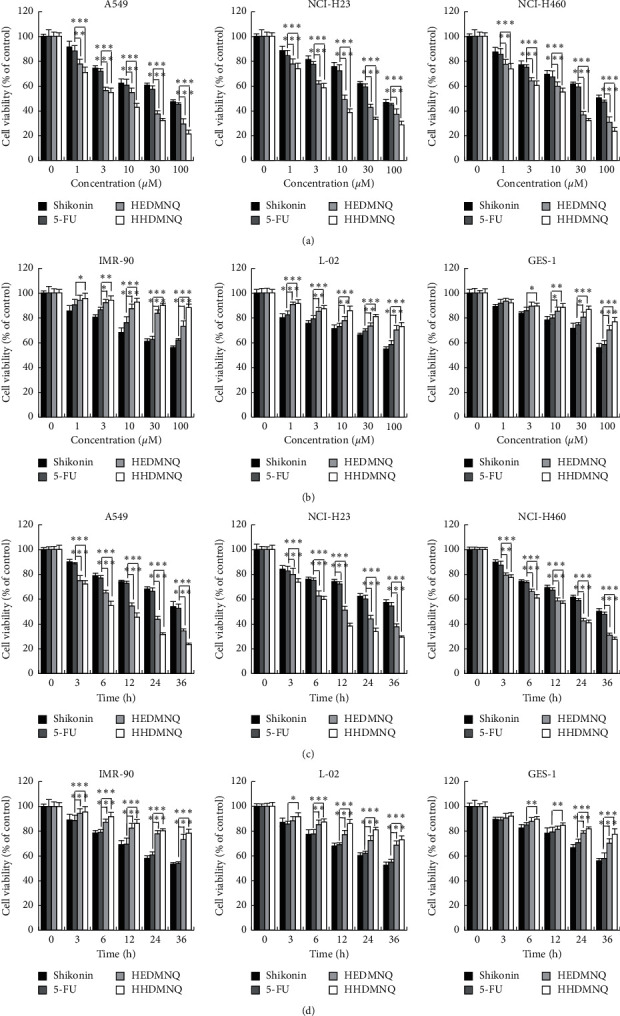
Effects of HEDMNQ and HHDMNQ on cell growth in human lung cancer cells. (a) Cell viabilities of A549, NCI-H23, and NCI-H460 cells. (b) Cell viabilities of normal lung IMR-90, liver L-02, and stomach GES-1 cells. Cells were seeded in 96-well plates and treated with different concentrations of shikonin, 5-FU, HEDMNQ, and HHDMNQ (0, 1, 3, 10, 30, and 100 *μ*M) for 24 h, and then the cell viabilities were determined by the CCK-8 assay. (c) Cell viabilities of A549, NCI-H23, and NCI-H460 cells. (d) Cell viabilities of normal lung IMR-90, liver L-02, and stomach GES-1 cells. Cells were seeded in 96-well plates and treated with shikonin, 5-FU, HEDMNQ, and HHDMNQ for 3, 6, 12, 24, and 36 h, and then the cell viabilities were determined by the CCK-8 assay. Data were collected from three individual experiments (^*∗*^*p* < 0.05, ^*∗∗*^*p* < 0.01, ^*∗∗∗*^*p* < 0.001).

To determine the cell cytotoxicity after HEDMNQ and HHDMNQ treatment, cell viabilities were measured by using the CCK-8 assay. As shown in Figures 2(a) and 2(c), HEDMNQ and HHDMNQ significantly inhibited the growth of A549, NCI-H23, and NCI-H460 non-small-cell lung cancer cells in a dose- and time-dependent manner, and the IC_50_ values of Shikonin, 5-FU, HEDMNQ, and HHDMNQ for each cell line are shown in Table 1. As shown in Figures 2(b) and 2(d), HEDMNQ and HHDMNQ had significantly lower cytotoxicity compared with shikonin and 5-FU in normal lung IMR-90, liver L-02, and stomach GES-1 cells. These data indicated that HEDMNQ and HHDMNQ selectively inhibited the viability of lung cancer cells with less cytotoxic effects on nonmalignant cells. In addition, HHDMNQ had the stronger inhibitory effects on cell viability, and compared with other cells, the A549 cells have a lower IC_50_ value and more sensitive to HHDMNQ, so we chose them for further study.

### 3.3. HHDMNQ Induces Cell Cycle Arrest at the G2/M Phase in A549 Cells

**Figure 3:**
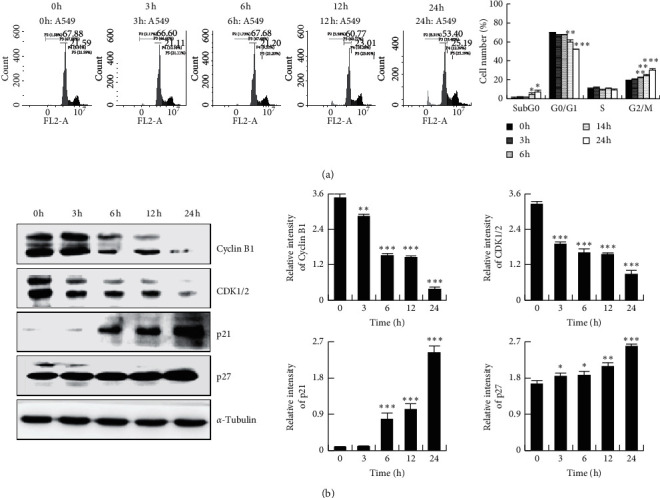
Effects of HHDMNQ treatment (3, 6, 12, and 24 h) on the A549 cell cycle. (a) Cells were stained with PI, and the cell cycle distribution was determined by flow cytometry and quantitative cell cycle analysis. (b) The expression levels of CDK1/2, cyclin B1, p21, and p27 proteins were analyzed by western blot and quantitative histograms of proteins (^*∗*^*p* < 0.05, ^*∗∗*^*p* < 0.01, ^*∗∗∗*^*p* < 0.001).

To assess the effect of HHDMNQ on cell cycle distribution, A549 cells were treated with HHDMNQ for 3, 6 12, and 24 h. The percentage of cells at the *G*_0_/*G*_1_ phase was decreased and that at the G2/M phase was increased in a time-dependent manner. Next, we detected the G2/M phase-related proteins to evaluate HHDMNQ-induced cell cycle arrest (Figure 3(a)). The expression levels of p21 and p27 were increased, and those of cyclin B1 and cyclin-dependent kinase 1/2 (CDK1/2) were decreased (Figure 3(b)). These results suggested that HHDMNQ arrested the cell cycle at the G2/M phase concomitantly with the downregulation of cyclin B1 and CDK1/2 in A549 cells.

### 3.4. HHDMNQ Induces Apoptosis in A549 Cells

**Figure 4:**
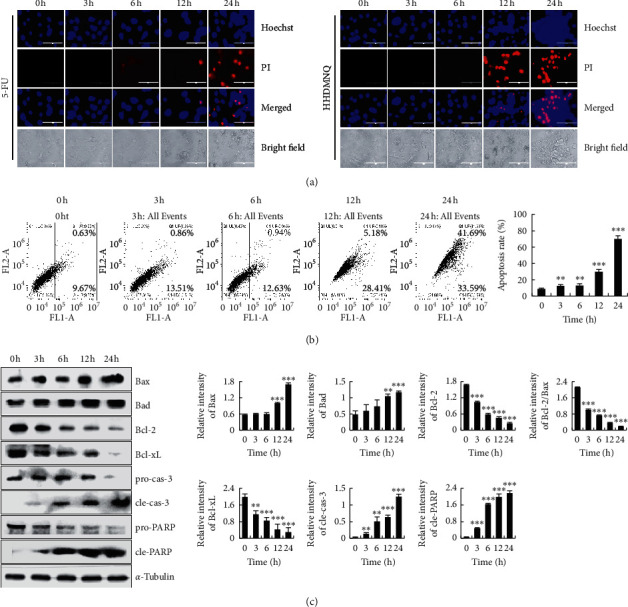
Effects of HHDMNQ on the morphological changes, apoptosis rate, and intrinsic apoptosis pathway in A549 cells. (a) A549 cells treated with 6 *μ*M 5-FU or HHDMNQ for 3, 6, 12, and 24 h fluorescence microscopic images (magnification, 200×) and relative fluorescence intensity. (b) Apoptosis distribution of A549 cells was determined by Annexin V-FITC/PI staining and quantification of early and late apoptotic cells. (c) A549 cells treated with 6 *μ*M HHDMNQ for 3, 6, 12, and 24 h. Expression levels of Bax, Bad, Bcl-2, Bcl-xL, pro-caspase-3, cleaved caspase-3, pro-PARP, cleaved PARP, and Bcl-2/Bax ratio were analyzed by western blot and quantitative histograms of proteins (^*∗*^*p* < 0.05, ^*∗∗*^*p* < 0.01, ^*∗∗∗*^*p* < 0.001).

To assess whether the suppressed cell viability effects of HHDMNQ were associated with apoptosis, A549 lung cancer cells were stained with Hoechst 33342 and PI. As shown in Figure 4(a), after treatment with HHDMNQ, nuclear chromatin condensation and fragmented punctuate blue and red nuclear fluorescence were observed in A549 cells in a time-dependent manner, similar to morphological changes in the apoptotic cells. To further assess the apoptosis caused by HHDMNQ, the apoptosis rate of A549 cells was examined by flow cytometry. HHDMNQ triggered the apoptosis of A549 cells, starting at 12 h, in a time-dependent manner (Figure 4(b)). These results suggested that HHDMNQ could induce the apoptosis of lung cancer A549 cells. Since HHDMNQ could induce apoptosis of A549 cells, we examined apoptosis-related proteins by western blot. As shown in Figure 4(c), the expression levels of Bcl-2-associated *X* protein (Bax), Bcl-2-associated death promoter (Bad), cleaved caspase-3, and cleaved PARP were increased after treatment with HHDNMQ, whereas the expression levels of the anti-apoptotic proteins B-cell lymphoma 2 (Bcl-2), B-cell lymphoma-extra-large (Bcl-xL), pro-caspase-3, pro-poly (ADP-ribose) polymerase (PARP), and Bcl-2/Bax ratio were significantly decreased in a time-dependent manner. These results illustrated that HHDMNQ activated the mitochondria-dependent pathway to induce apoptosis in A549 lung cancer cells.

### 3.5. HHDMNQ Regulates the MAPK, STAT3, and NF-κB Signalling Pathways in A549 Cells

**Figure 5:**
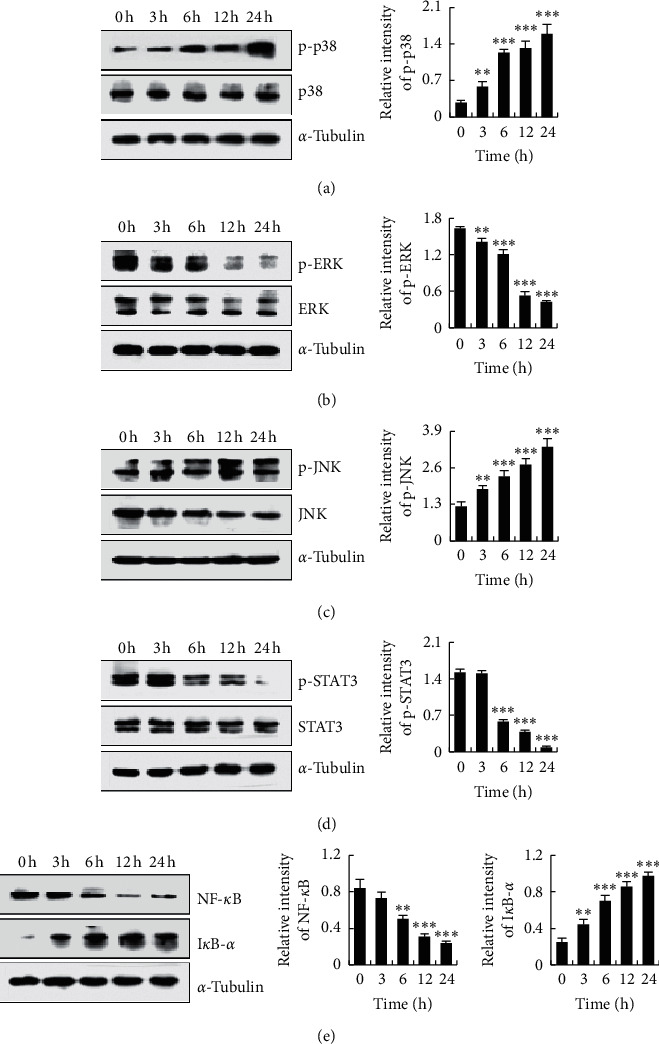
Effects of HHDMNQ on the MAPK, STAT3, and NF-*κ*B signalling pathways in A549 cells. (a–e) A549 cells treated with 6 *μ*M HHDMNQ for 3, 6, 12, and 24 h. Expression levels of p38, ERK, JNK, STAT3, NF-*κ*B (p65), and I*κ*B-*α* were analyzed by western blot and quantification histograms of proteins (^*∗*^*p* < 0.05, ^*∗∗*^*p* < 0.01, ^*∗∗∗*^*p* < 0.001).

To investigate the underlying mechanisms of HHDMNQ-induced apoptosis in lung cancer cells, the key proteins in the MAPK, STAT3, and NF-*κ*B signalling pathways were examined. As shown in Figures 5(a)–5(e), HHDMNQ treatment inhibited the activation of p-ERK, p-STAT3 (Tyr 705) and NF-*κ*B (p65) but promoted the expression of p-p38, p-JNK, and I*κ*B-*α* in a time-dependent manner. These results indicated that regulating MAPK, STAT3, and NF-*κ*B activity may promote HHDMNQ-mediated induction of cell apoptosis in lung cancer.

### 3.6. HHDMNQ Induces Oxidative Stress-Mediated Apoptosis in A549 Cells

**Figure 6:**
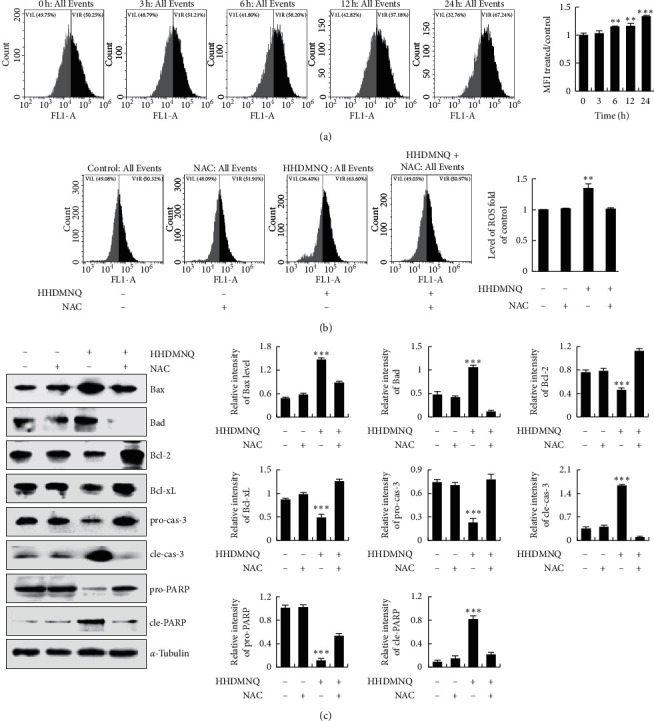
Effects of HHDMNQ on intracellular ROS levels and mitochondrial pathways in A549 cells. (a) A549 cells treated with 6 *μ*M HHDMNQ for 3, 6, 12, and 24 h, and intracellular ROS levels were measured by flow cytometry and quantification histograms of intracellular ROS levels. (b) A549 cells treated with or without NAC for 24 h, and intracellular ROS levels were measured by flow cytometry and quantification histograms of intracellular ROS levels. (c) A549 cells treated with or without NAC for 24 h; expression levels of Bax, Bad, Bcl-2, Bcl-xL, caspase-3, and PARP were measured by western blot and quantitative histograms of proteins (^*∗*^*p* < 0.05, ^*∗∗*^*p* < 0.01, ^*∗∗∗*^*p* < 0.001).

To determine whether intracellular ROS levels are associated with activation of the programmed cell apoptosis mechanism in lung cancer cells, the levels of ROS and cell apoptosis were determined. As shown in Figure 6(a), the levels of ROS were significantly increased in a time-dependent manner after treating A549 cells with HHDMNQ for 3, 6, 12, and 24 h. As shown in Figure 6(b), intracellular ROS levels were suppressed after pretreatment with the specific ROS inhibitor NAC. To demonstrate whether HHDMNQ-induced increases in ROS were associated with the apoptosis of A549 cells, cell apoptosis-related proteins were determined. As shown in Figure 6(c), NAC significantly reversed the increased expression levels of Bax, Bad, cleaved caspase-3, and cleaved PARP and the decreased the expression levels of Bcl-2, Bcl-xL, pro-caspase-3, and pro-PARP. These findings demonstrated that HHDMNQ increased intracellular ROS levels, resulting in A549 cell apoptosis.

### 3.7. HHDMNQ Induces Apoptosis through ROS-Mediated MAPK, STAT3, and NF-*κ*B Signalling Pathways in A549 Cells

**Figure 7:**
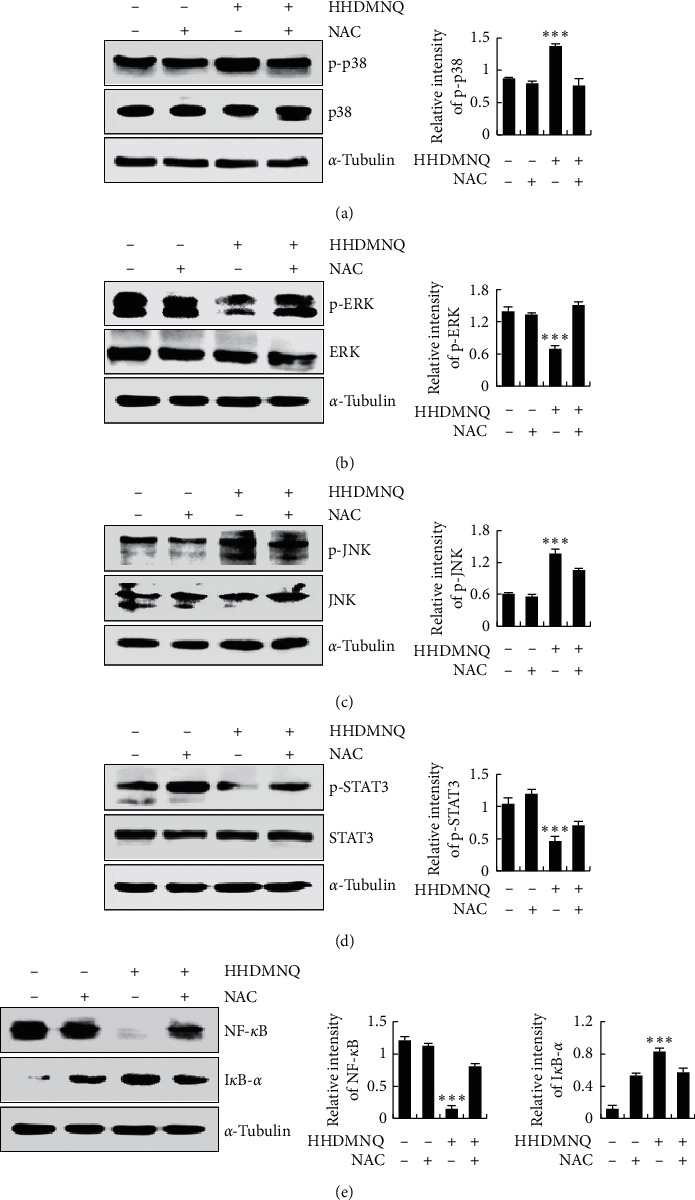
Effects of NAC on HHDMNQ-induced oxidative stress for apoptosis in A549 cells. (a–e) Expression levels of p38, ERK, JNK, STAT3, NF-*κ*B, and I*κ*B-*α *were analyzed by western blot and quantitative histograms of proteins (^*∗*^*p* < 0.05, ^*∗∗*^*p* < 0.01, ^*∗∗∗*^*p* < 0.001).

To demonstrate the underlying molecular mechanism of HHDMNQ-induced ROS-mediated apoptosis of A549 cells, we examined the effects of HHDMNQ on MAPK, STAT3, and NF-*κ*B signalling pathways that play pivotal roles in cancer cells. As shown in Figures 7(a)–7(e), the expression levels of p38, JNK, and I*κ*B-*α* were increased and those of ERK, STAT3, and NF-*κ*B were decreased after HHDMNQ treatment. However, the expression levels of proteins were reversed in the HHDMNQ and NAC treatment groups. These results indicated that HHDMNQ induced A549 cell apoptosis by regulating ROS-mediated MAPK, STAT3, and NF-*κ*B signalling pathways.

## 4. Discussion

There is an urgent need to develop novel anticancer drugs, and inhibition of proliferation and induction of apoptosis in cancer cells are the potential therapeutic strategies for drug development [24]. The 1,4-naphthoquinone pharmacophore has growth inhibitory activity and can induce apoptosis and anti-inflammation in various cancer cell lines. Furthermore, the side chain can influence the entire activity of 1,4-naphthoquinone derivatives [25]. In this study, we used 1,4-naphthoquinone as a pharmacophore to synthesize the novel naphthoquinone derivatives HEDMNQ and HHDMNQ. The 1,4-naphthoquinone derivatives suppressed the growth of lung cancer cells with the lower IC_50_ values and lower cytotoxicity in normal cell lines. HHDMNQ had higher efficiency and lower cytotoxicity than HEDMNQ. Furthermore, HHDMNQ induced cell cycle arrest at the G2/M phase by regulating the CDK/cyclin complex in A549 lung cancer cells.

Induction of cell apoptosis is a crucial physiological process to inhibit cancer cell proliferation. In addition, cell apoptosis is related to the promotion of pro-apoptotic proteins and the suppression of anti-apoptotic proteins, and the Bcl-2 family is foremost in regulating the mitochondrial pathway [26]. In this study, fluorescence microscopy showed A549 cell shrinkage, deformation, and shedding; and flow cytometry results showed that early apoptotic and late apoptotic cells were increased after HHDMNQ treatment in a time-dependent manner. Furthermore, HHDMNQ promoted the downregulation of Bcl-2 and Bcl-xL and the upregulation of Bad, Bax, cleaved caspase-3, and cleaved PARP in A549 cells. Thus, it can be considered that HHDMNQ-mediated induction of A549 lung cancer cell apoptosis is associated with regulation of the mitochondria-regulated apoptosis pathway. In addition, some studies suggested that the mutant p53 could promote cancer cell proliferation, invasion, and drug resistance, and the wild-type p53 could mediate various cellular activities, such as promote cell cycle arrest and apoptosis by inhibiting the phosphorylation of STAT3 and regulating the expression of cell cycle target genes [27, 28]. It has been shown that 2-(naphthalene-2-thio)-5,8-dimethoxy-1,4-naphthoquinone could induce G0/G1 phase arrest by regulating the wild-type p53 in cancer cells [29]. The structures of 2-(naphthalene-2-thio)-5,8-dimethoxy-1,4-naphthoquinone are similar to the compound that we synthesized. On the basis of the above, we speculate that HHDMNQ induced cell apoptosis and G2/M cell cycle arrest may be related to p53 expression.

Developing anticancer agents that modulate inflammatory signalling pathways and cell proliferation may be a rational strategy for preventing or treating lung cancers. It has been demonstrated that ERK, JNK, and p38 are the three main components of the MAPK signalling pathway, and they play an important regulatory role in cancer cell apoptosis [30]. ERK is a key protein kinase in the MAPK family, whose abnormal activation will cause the proliferation and invasion of various tumor cells. The results indicated that paxillin promoted Bcl-2 activation by mediating ERK activation, which was associated with tumor formation efficacy in mice [31]. The Janus kinase (JAK)/STAT signalling pathway is one of the important signalling pathways in modulating cell proliferation, differentiation, migration, and apoptosis [32]. STAT3 maintains an abnormal activation state, which can accelerate the cycle process by controlling the protein levels of cyclin *D*, p21, and p27, promoting the new blood vessels generation and providing supplementary nutrition for the development of cancer cells. Some studies suggested that celecoxib induced apoptosis and cell cycle arrest via inhibition of STAT3 phosphorylation in NPC cells [33, 34]. The NF-*κ*B signalling pathway is associated with the growth and survival of cancer cells [35]. I*κ*B-*α* is an important factor in the NF-*κ*B family of inhibitors, whose phosphorylation is closely related to NF-*κ*B activation. The phosphorylation of I*κ*B-*α* will result in the ubiquitin-proteasome system degradation, producing amounts of NF-*κ*B dimers, while inhibition of the phosphorylation leads to the inactivation of NF-*κ*B. And the inhibition effects on NF-*κ*B activation caused the ratio of Bax/Bcl-2 upregulated in the Bcl-2 family and then promoted the activation of caspase-3 [36, 37]. In this study, we examined whether HHDMNQ treatment regulates the MAPK, STAT3, and NF-*κ*B signalling pathways. We found that HHDMNQ efficiently promoted the phosphorylation of p38 and JNK and inhibited the phosphorylation of ERK, STAT3, NF-*κ*B and I*κ*B-*α* in a time-dependent manner. The MAPK, STAT3 and NF-*κ*B signalling pathways were inhibited by HHDMNQ, and they were closely associated with the suppression of proliferation and induction of A549 lung cancer cell apoptosis.

Oxidative stress regulates cancer cell apoptosis by activating multifarious redox signalling cascade reactions [38]. The mitochondrial pathway is the main component of the intrinsic apoptosis mechanism, and it is the main target of ROS. The Bcl-2 family of proteins and caspases is the characteristic feature of ROS-mediated mitochondrial-induced apoptosis in cancer cells [39]. Numerous small-molecule compounds have been shown to stimulate excessive ROS production, and they are associated with the induction of cell apoptosis. In this study, HHDMNQ promoted the accumulation of ROS in A549 cells in a time-dependent manner, and pretreatment with NAC restored the levels of ROS. HHDMNQ induced cell apoptosis, and the expression levels of apoptosis regulators were completely reversed by NAC pretreatment. We hypothesized that HHDMNQ could modulate MAPK, STAT3, and NF-*κ*B signalling pathway activation through regulating intracellular ROS levels to induce A549 cell apoptosis. We confirmed our hypotheses by using NAC, which reversed the expression levels of MAPK, STAT3, and NF-*κ*B signalling-pathway-related proteins in A549 cells. Pretreatment of A549 cells with NAC not only abolished the effects of HHDMNQ-induced apoptosis but also reversed HHDMNQ-mediated suppressive effects on MAPK, STAT3, and NF-*κ*B signalling pathways in A549 cells.

## 5. Conclusion

**Figure 8:**
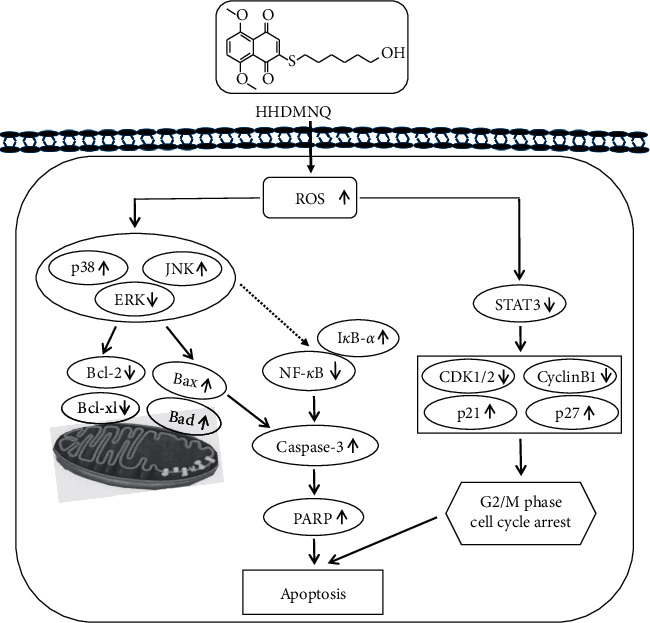
Schematic mechanisms illustration of HHDMNQ-induced apoptosis in lung cancer A549 cells. The molecular mechanisms of HHDMNQ anticancer activity as related to the ROS generation and MAPK/STAT3/NF-*κ*B pathways.

In conclusion, we synthesized the two novel types of 1,4-naphthoquinone derivatives HEDMNQ and HHDMNQ. This study revealed for the first time that HHDMNQ induced mitochondria-dependent apoptosis and G2/M cell cycle arrest promoted the accumulation of ROS and mediated MAPK/STAT3/NF-*κ*B signalling pathways and then regulated the downstream-related apoptosis and cycle proteins in A549 lung cancer cells (Figure 8).

## Figures and Tables

**Table 1:** IC_50_ values of shikonin, 5-FU, HEDMNQ, and HHDMNQ in lung cancer cells.

Cell name	Shikonin (*μ*M)	5-FU (*μ*M)	HEDMNQ (*μ*M)	HHDMNQ (*μ*M)
A549	52.83 ± 2.02	52.83 ± 2.02	10.91 ± 1.22	5.62 ± 2.16
NCI-H23	71.55 ± 2.35	69.31 ± 3.39	14.78 ± 2.31	6.62 ± 1.65
NCI-H460	79.19 ± 2.12	74.14 ± 1.84	14.22 ± 2.06	9.07 ± 1.38

## Data Availability

The data used to support the findings of this study are available from the corresponding author upon request.
